# Refractory Hemorrhage Following Total Knee Replacement Unmasking Acquired Hemophilia A

**DOI:** 10.7759/cureus.110166

**Published:** 2026-06-03

**Authors:** Ravindrakumar Manickam, Neavean Raj Anandan, Sasidaran Ramalingam, Kishandh T Harikrishnan, Abdul Muttalib Bin Abdul Wahid

**Affiliations:** 1 Department of Orthopaedics and Traumatology, Hospital Tuanku Ja'afar, Seremban, Seremban, MYS

**Keywords:** acquired hemophilia a (aha), activated partial thromboplastin time (aptt), arthoplasty, postoperative bleeding, total knee replacement (tkr)

## Abstract

Acquired hemophilia A (AHA) is a rare autoimmune bleeding disorder caused by autoantibodies against factor VIII and may present with life-threatening hemorrhage. Postoperative presentation is uncommon and may lead to delayed diagnosis, as bleeding is often initially attributed to surgical causes.

We report a 64-year-old man with post-traumatic knee arthritis and retained hardware who underwent navigation-assisted total knee replacement. The procedure was uneventful with an estimated blood loss of 300 mL. On postoperative day two, he developed acute knee swelling with persistent bleeding through a negative-pressure wound dressing. Urgent re-exploration and hematoma evacuation were performed; however, diffuse bleeding persisted despite adequate surgical hemostasis, requiring activation of a massive transfusion protocol.

Laboratory investigations demonstrated isolated prolongation of activated partial thromboplastin time (aPTT) at 65.3 seconds. Further coagulation studies revealed markedly reduced factor VIII activity with circulating factor VIII inhibitors, confirming AHA. Hemostasis was achieved following treatment with FEIBA and NovoSeven under hematology guidance. The patient subsequently recovered well and was discharged with outpatient follow-up.

This case highlights acquired hemophilia A as a rare but important differential diagnosis in persistent postoperative bleeding following total knee replacement. Early recognition, prompt coagulation workup, and multidisciplinary management are essential to improve outcomes.

## Introduction

Acquired hemophilia A (AHA) is a rare autoimmune bleeding disorder caused by autoantibodies against factor VIII, with an incidence of approximately 1-1.5 cases per million annually [1]. It can present with life-threatening hemorrhage and may pose a diagnostic challenge in the postoperative setting, where bleeding is often initially attributed to surgical causes [2].

## Case presentation

A 64-year-old gentleman with post-traumatic knee arthritis and retained hardware underwent navigation-assisted total knee replacement. The procedure was uneventful, with an estimated blood loss of 300 mL.

On postoperative day two, he developed acute knee swelling and persistent bleeding through a negative-pressure wound dressing. Urgent re-exploration with hematoma evacuation and surgical hemostasis was performed; however, diffuse bleeding persisted, necessitating activation of a massive transfusion protocol.

Laboratory evaluation demonstrated an isolated prolonged activated partial thromboplastin time (aPTT) of 65.3 seconds (reference range 27.9-38.7 seconds) (Table 1). Subsequent coagulation studies revealed markedly reduced factor VIII activity with circulating factor VIII inhibitors, confirming AHA.

**Table 1:** Coagulation studies taken for the patient showing elevated levels of APTT and factor VIII inhibitor with reduced factor VIII levels PT: prothrombin time, INR: international normalized ratio, APTT: activated partial thromboplastin time

Parameter	Result	Unit	Reference Range
PT	11.1	Seconds	9.8-12
INR	1.02	Ratio	0.8-1.2
aPTT	65.3	Seconds	27.9-38.7
Single Factor VIII Assay	13.21	%	50-150
Factor VIII inhibitor	38.4	BU/ml	<0.5

Hemostatic control was achieved following treatment with FEIBA (anti-inhibitor coagulant complex) and NovoSeven (recombinant activated factor VII), which are recommended first-line bypassing agents for active bleeding in AHA patients with inhibitors [2,3].

In addition to hemostatic management with bypassing agents, the patient was started on intravenous hydrocortisone and oral cyclophosphamide throughout admission as immunosuppressive therapy for inhibitor eradication.

**Figure 1:**
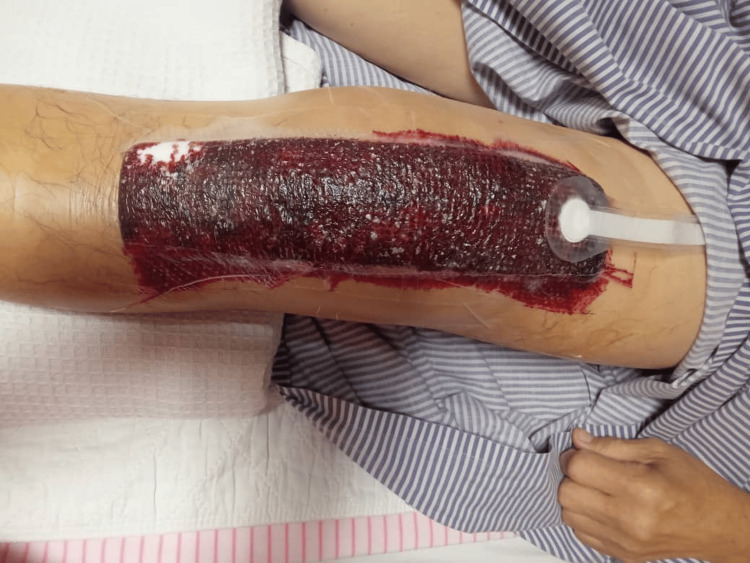
Postoperative bleeding through negative pressure wound dressing

**Figure 2:**
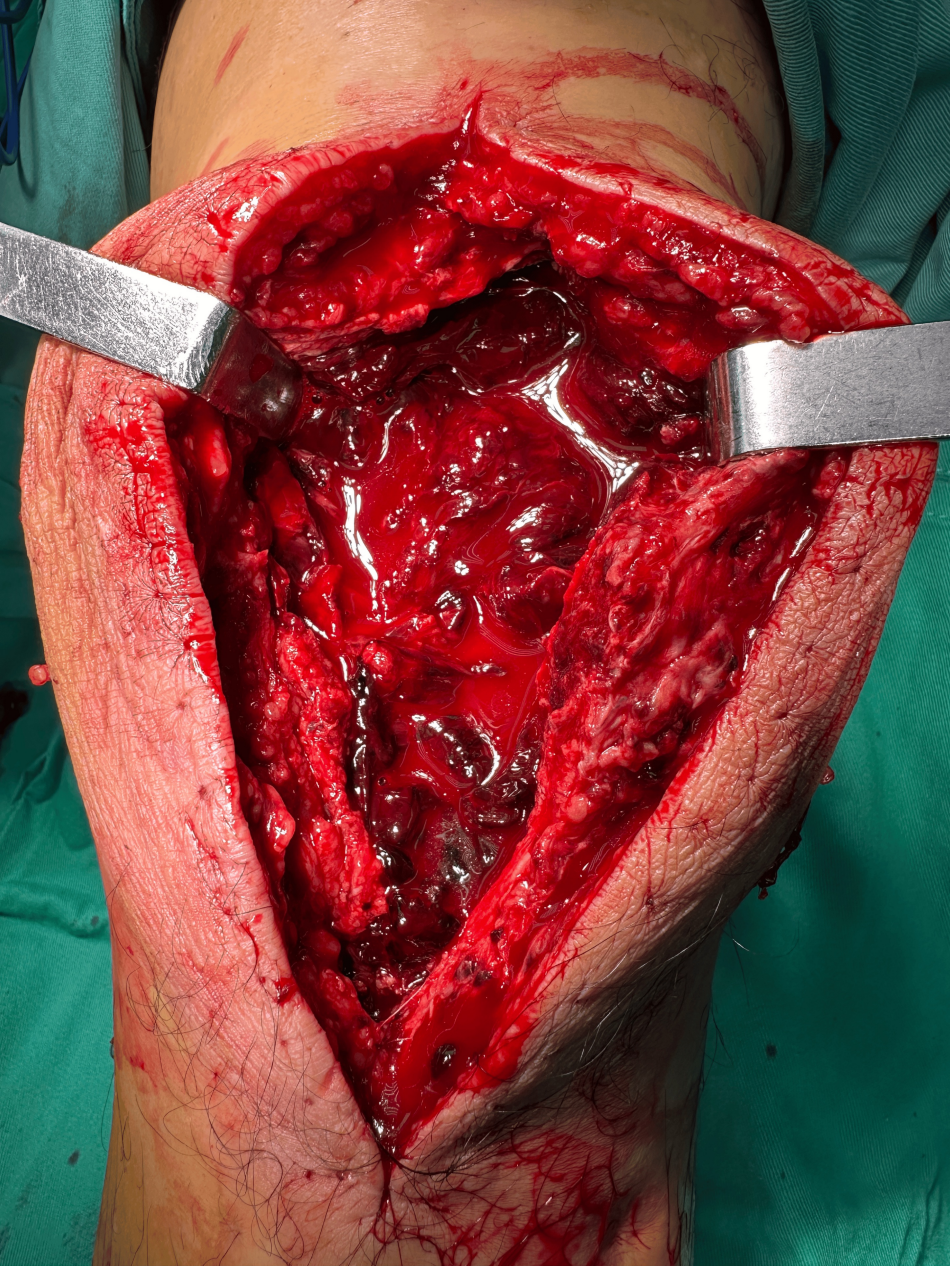
Intraoperative findings during second-look surgery demonstrating hematoma

The patient recovered well and was discharged with ongoing specialist follow-up. Figure 1 shows the postoperative bleeding through negative pressure wound dressing. Figure 2 shows the intraoperative findings during second-look surgery demonstrating hematoma

## Discussion

AHA is a rare autoimmune coagulopathy caused by autoantibodies directed against factor VIII, resulting in impaired intrinsic coagulation and potentially life-threatening bleeding. The condition predominantly affects older adults, with a median age of presentation between 60 and 80 years, and approximately half of the cases are idiopathic. The remaining cases are associated with autoimmune disorders, malignancy, pregnancy, infections, or certain medications [1,3]. The estimated annual incidence is approximately 1 to 1.5 cases per million population, contributing to frequent delays in recognition due to its rarity [1].

Clinically, AHA differs significantly from congenital hemophilia. While congenital hemophilia commonly presents with recurrent hemarthrosis, AHA more frequently manifests as spontaneous soft tissue bleeding, muscle hematomas, gastrointestinal hemorrhage, genitourinary bleeding, or mucosal bleeding. Data from the European Acquired Haemophilia Registry (EACH2) demonstrated that subcutaneous bleeding occurred in nearly 80% of patients, whereas joint bleeding was uncommon [4]. This makes our case particularly unusual, as the patient initially presented with persistent postoperative hemarthrosis following total knee replacement, a scenario that can easily be mistaken for surgical bleeding complications such as vessel injury, inadequate intraoperative hemostasis, or anticoagulant-related bleeding.

The diagnostic challenge in postoperative patients lies in the tendency to attribute persistent bleeding to surgical causes, often resulting in repeated procedures before an underlying hematological disorder is considered. In our patient, persistent hemorrhage despite re-exploration and adequate surgical hemostasis raised suspicion for an alternative etiology. A hallmark laboratory finding in AHA is isolated prolongation of activated partial thromboplastin time (aPTT) with normal prothrombin time (PT) and international normalized ratio (INR), as demonstrated in this case. Current international guidelines recommend prompt mixing studies in patients with unexplained isolated prolonged aPTT, followed by factor VIII activity assays and Bethesda inhibitor testing to confirm the diagnosis [2]. Early recognition is crucial because delays in diagnosis have been associated with increased transfusion requirements, prolonged hospitalization, and increased mortality.

Mortality in AHA remains substantial, with reported rates ranging from 9% to 22%, primarily due to severe hemorrhage, complications of immunosuppressive therapy, and delayed diagnosis [3,5]. Hemostatic treatment aims to control acute bleeding using bypassing agents such as activated prothrombin complex concentrate (FEIBA) or recombinant activated factor VII (NovoSeven), both of which are recommended as first-line therapy in patients with high inhibitor titers [2]. Recombinant porcine factor VIII has also emerged as an effective treatment option in selected settings, although availability remains limited in many institutions [3].

In addition to bleeding control, eradication of inhibitors through immunosuppressive therapy remains a cornerstone of treatment. Common regimens include corticosteroids alone or in combination with cyclophosphamide or rituximab, depending on factor VIII levels, inhibitor titers, and patient comorbidities [2,6]. Although our report focuses primarily on acute orthopedic presentation, long-term hematological follow-up remains essential to monitor inhibitor eradication and prevent recurrence.

Reports of AHA presenting after orthopedic procedures remain exceedingly rare in the literature. Most published cases involve spontaneous bleeding episodes or minor procedural interventions rather than major joint arthroplasty. Postoperative bleeding following total knee arthroplasty has been shown to negatively affect functional outcomes, increase complication rates, and prolong recovery in patients with underlying bleeding disorders [7]. This case highlights how persistent postoperative bleeding following total knee replacement may represent the first manifestation of an underlying acquired coagulation disorder. For orthopedic surgeons, maintaining awareness of AHA is critical when postoperative bleeding appears disproportionate to intraoperative findings or fails to respond to conventional surgical management.

## Conclusions

This case highlights acquired hemophilia A as a rare but potentially life-threatening cause of persistent postoperative bleeding following total knee replacement. The unusual presentation as refractory hemarthrosis after an otherwise uncomplicated arthroplasty emphasizes the importance of maintaining a high index of suspicion when bleeding persists despite adequate surgical intervention. Early recognition of isolated prolonged aPTT, timely hematological investigations, and prompt initiation of appropriate hemostatic therapy are essential to prevent unnecessary reoperations, reduce morbidity, and improve patient outcomes. Greater awareness among orthopedic surgeons may facilitate earlier diagnosis of this uncommon but serious condition.
